# Importance and Characterisation of Concurrent Pathogens in Diarrhoeic Calves from North-Western Spain

**DOI:** 10.3390/ani15182735

**Published:** 2025-09-19

**Authors:** Cynthia López-Novo, Pablo Díaz, José Manuel Díaz-Cao, Seila Couso-Pérez, David García-Dios, Gonzalo López-Lorenzo, Susana Remesar, Elvira Ares-Mazás, Patrocinio Morrondo, Hipólito Gómez-Couso, Alberto Prieto

**Affiliations:** 1 Investigación en Sanidad Animal: Galicia (Grupo INVESAGA), Departamento de Patología Animal, Facultad de Veterinaria, Universidade de Santiago de Compostela, Campus Terra, Avd. Carballo Calero s/n, 27002 Lugo, Spain; cynthia.lopez.novo@usc.es (C.L.-N.); josemanueldiaz.cao@usc.es (J.M.D.-C.); david.garcia.dios@rai.usc.es (D.G.-D.); gonzalo.lopezlorenzo@usc.es (G.L.-L.); susana.remesar@usc.es (S.R.); patrocinio.morrondo@usc.es (P.M.); alberto.prieto@usc.es (A.P.); 2 Grupo Interdisciplinar en Tecnología Farmacéutica, Inmunobiología Parasitaria y Parasitosis Hídricas (PARAQUASIL), Departamento de Microbiología y Parasitología, Facultad de Farmacia, Universidade de Santiago de Compostela, Campus Vida, 15782 Santiago de Compostela, Spain; seila.couso.perez@usc.es (S.C.-P.); melvira.ares@usc.es (E.A.-M.); hipolito.gomez@usc.es (H.G.-C.); 3 Instituto de Investigación del Medio Acuático para Una Salud Global (iARCUS), Universidade de Santiago de Compostela, Campus Vida, 15782 Santiago de Compostela, Spain; 4 Instituto de Biodiversidade Agraria e Desenvolvemento Rural (IBADER), Universidade de Santiago de Compostela, Campus Terra, 27002 Lugo, Spain

**Keywords:** neonatal calf diarrhoea, cattle, enteropathogens, co-occurrence

## Abstract

Neonatal calf diarrhoea (NCD) is the leading cause of mortality in calves under 30 days old. Although several pathogens are usually involved in outbreaks, most previous research has focused on specific enteropathogens or on the four agents traditionally associated with this syndrome. This study aimed to determine the prevalence of thirteen enteropathogens in 420 diarrhoeic calves under a month of age from north-western Spain and to identify the most frequent co-occurrences. Four diarrhoeagenic pathotypes of *Escherichia coli*, bovine rotavirus A, coronavirus, norovirus, nebovirus and torovirus were identified using qPCR. Presence of *Salmonella* spp. was studied using a serum agglutination test. A direct immunofluorescence assay was employed to identify *Cryptosporidium* and *Giardia duodenalis* oo/cysts. *Eimeria* oocysts were found by the floatation technique. Our results confirm the co-occurrence of agents in most cases of NCD. A total of 172 pathogen combinations, involving up to seven agents, were identified. Cases involving one or more viral agents and *Cryptosporidium parvum* were predominant, maybe because viral immunosuppression facilitates the protozoan life-cycle. The frequent detection of agents typically overlooked in the aetiological diagnosis of NCD suggests that they may play a role in this syndrome and thus should be included in routine diagnostic panels, allowing implementing the most effective control measures.

## Simple Summary

Neonatal calf diarrhoea (NCD) is a common syndrome affecting newborn calves, leading to significant economic losses. Several bacterial, viral and parasitic agents can be involved in the onset of NCD. Mixed infections are common, often resulting in more severe cases. However, most studies have primarily focused on particular agents or, at most, the four enteropathogens traditionally associated with this syndrome (enterotoxigenic *Escherichia coli*, bovine rotavirus A, bovine coronavirus and *Cryptosporidium parvum*). Consequently, information on other agents linked to NCD is limited, hindering the implementation of the most effective control measures. The aim of this study was to determine the prevalence and the most frequent associations of thirteen enteropathogens in diarrhoeic calves under a month of age from north-western Spain. Our results reveal that the co-occurrence of two to four enteropathogens was the most common. In fact, up to seven different agents were identified in a single sample. Moreover, several pathogens not typically included in routine diagnostic panels for NCD were detected quite frequently, suggesting that their inclusion may improve the aetiological diagnosis of this syndrome. Achieving an accurate diagnosis is key for implementing the most effective control measures, including specific treatments, and reducing the impact of NCD in cattle farms.

## 1. Introduction

Scientific progress and innovative technologies have noticeably transformed the cattle sector in recent years. Improvements in areas such as nutrition, animal welfare, herd management, host genetics, and reproductive technologies have enhanced productivity, efficiency and profitability, leading to a better detection and prevention of pathologies [1,2,3]. Despite all these advancements, neonatal calf diarrhoea (NCD) is still a common condition in calves under one month of age, constituting the major cause of morbidity and mortality in these animals and leading to substantial economic losses [1]. The frequent occurrence of NCD in cattle farms is mainly related to its multi-factorial nature which involves the interaction among several factors such as the immune status of the calf or the environmental infection pressure [2,3]. In addition, the aetiological component plays a key role in this syndrome. Traditionally, the main enteropathogens associated with NCD have been enterotoxigenic *Escherichia coli* (ETEC), bovine rotavirus A (BRoV), bovine coronavirus (BCoV) and *Cryptosporidium parvum* [4,5]. Other agents such as *Eimeria* spp., *Giardia duodenalis*, bovine viral diarrhoea virus (BVDV) and *Salmonella* spp. have been frequently related to NCD; in addition, other pathotypes of *E. coli* (enteropathogenic [EPEC], Shiga-toxin producing [STEC] and enterohaemorrhagic [EHEC]) and emergent viruses, including bovine torovirus (BToV), bovine norovirus (BNoV), bovine nebovirus (BNeV) or bovine astrovirus (BoAstV), have also been involved in this syndrome since they have been frequently identified in diarrhoeic calves, but their relationship with NCD is still controversial [1,2,6,7,8,9,10,11,12]. In this regard, it is worth noting that most investigations on this syndrome have focused on the presence of specific pathogens, and most of the few studies considering multiple agents only analyse the presence of the four traditionally associated with NCD. However, those investigations including several enteropathogens prove that the co-occurrence of pathogens is common in diarrhoeic calves, with prevalences between 23.5% and 90.0% [11,13,14,15,16,17,18,19,20,21], being commonly associated with more severe clinical signs [11,22].

Although several studies regarding the aetiology of NCD have been conducted in Spain, they are mainly focused on the prevalence of the four agents traditionally associated with this syndrome [18,20,21,23]. Thus, there is limited and outdated information on the presence of other enteropathogens such as emergent viruses and diarrhoeagenic *E. coli* pathotypes different from ETEC [24,25]. Data available on this issue is also limited globally. For all these reasons, this study aims to determine the prevalence of thirteen enteropathogens in calves under a month of age from north-western Spain, including the four agents traditionally involved in NCD as well as other diarrhoeagenic pathotypes of *E. coli* (EPEC, STEC and EHEC), *Salmonella* spp., *G. duodenalis*, *Eimeria* spp., BToV, BNoV and BNeV. The significance of the concomitant presence of pathogens was also assessed, and the most common associations were further identified.

## 2. Materials and Methods

### 2.1. Study Design and Sample Collection

The study was performed in Galicia, north-western Spain, one of the most important cattle rearing areas in the country. In this region, most cattle herds are small: approximately, half of the farms have less of 9 animals, and the average size of animals per farm was 19.9. Cattle are mainly managed in semi-extensive rearing systems [26].

The minimum number of samples required for this investigation was calculated using Epitools (https://epitools.ausvet.com.au, accessed on 3 September 2025), considering a 95% confidence interval and 95% precision. Since data on the prevalence of several of the studied pathogens was not available in diarrhoeic calves from Spain, a 50% prevalence was considered, leading to the largest sampling size. Thus, the estimated minimum sample size was 385 animals.

The sampling was performed by 26 clinical veterinarians, which altogether covered all the study area. These practitioners were asked to collect faecal samples from diarrhoeic calves up to 30 days old in those farms they routinely visited. Calves treated with antibiotics, as stated by the submitting veterinarians, or showing other serious illnesses, such as pneumonia, were excluded. Complete specific guidelines were provided to each collaborating veterinarian before collection of samples in order to use the same criteria in all farms. A calf was considered diarrhoeic when its faecal consistency was semi-liquid or watery [27]; the faecal consistency was confirmed in the laboratory. The samples were collected directly from the rectum of the calves using both sterile containers and AMIES transport media swabs. For each sample, the age of the animal and the vaccination status of the farm of origin against BRoV, ETEC and BCoV were recorded, along with whether the animal had received halofuginone lactate prior to sampling. Data on the vaccines available is summarized in Supplementary Table S1. Samples were stored at 4 °C until examination, which mainly took place 24 h after sampling and no later than 48 h.

Finally, 420 samples from 222 farms were collected from May 2017 to June 2020. Most samples (47.1%; 198/420) were collected from calves younger than one week old, 29.3% (123/420) from animals between 8 and 14 days of age, 12.6% (53/420) from calves in their third week of life and 11% (46/420) from animals in their fourth week of age. No more than 11 calves were sampled in a single farm and most farms only contributed with four or fewer samples. A subset of these samples (404 out of 420) was previously included in a study mainly focused on the interactions among parasitic agents and their zoonotic repercussions. Thus, it is independent from the findings of this study [28].

### 2.2. Detection of Pathogens

All the faecal samples were tested for thirteen enteropathogens reported to be involved in calf diarrhoea: ETEC, EPEC, STEC, EHEC, *Salmonella* spp., BRoV, BCoV, *C. parvum*, *G. duodenalis*, *Eimeria* spp. and the emergent viruses of which the most epidemiological data was available (BToV, BNoV and BNeV). BVDV was not included since most farms in the study area belong to Health Defence Associations and have implemented a health programme for preventing and controlling different infectious diseases, including BVD.

For bacterial isolation, the swab was streaked onto MacConkey and Xylose Lysine Deoxycholate (XLD) agars and incubated at 37 °C for 24 h. The bacterial DNA of colonies compatible with *E. coli* was extracted using the boiling method [29] and the presence of five virulence factors (*eae*, F5, STa, Stx1, Stx2) was analysed by qPCR (EXOOne qPCR kits, Exopol, Zaragoza, Spain), following the manufacturer’s instructions. These qPCR kits contain, as endogenous control, a specific set of primers and probe for the glutamate decarboxylase (GAD) gene of *E. coli* strains, allowing confirmation that the amplified DNA was indeed that of *E. coli*. Consecutively, the presence of diarrhoeagenic *E. coli* pathotypes was determined for each sample: ETEC (F5 + Sta), EPEC (*eae*), STEC (Stx) and EHEC (*eae* + Stx) [8,30]. The presence of *Salmonella* spp. was confirmed in those samples showing compatible colonies using a commercial serum agglutination test (*Salmonella* Antisera, PRO-LAB Diagnostics, Bromborough, UK).

The detection of the viral agents (BRoV, BCoV, BToV, BNoV and BNeV) was performed by several commercial RT-qPCR kits (EXOOne qPCR kits, Exopol), following the manufacturer’s instructions, preceded by the extraction of the viral RNA directly from the faecal samples using a commercial RNA extraction kit (RNeasy Power Microbiome Kit, QIAGEN GmbH, Hilden, Germany).

All the qPCR and RT-qPCR reactions were run in an Applied Biosystems ABI Prism 7500 thermocycler (Thermo Fisher Scientific, Waltham, MA, USA). A synthetic control and molecular grade water were used as positive and negative controls, respectively. A sample was considered positive when the cycle threshold (Ct) was equal to or lower than 38.

Regarding parasites, *Cryptosporidium* spp. oocysts and *G. duodenalis* cysts were detected using a commercial immunofluorescence technique (Aqua-Glo G/C, Waterborne Inc., New Orleans, LA, USA), following the manufacturer’s instructions. To increase the sensitivity of this method, faecal samples were previously concentrated from 2 g of faeces using a diphasic (water/ethyl acetate) sedimentation method [31]. All the samples positive to *Cryptosporidium* spp. were genotyped at the small ribosomal subunit RNA (*18S rRNA*) gene as previously described [28]. Only those samples identified as *C. parvum* were included in this study. Finally, the presence of *Eimeria* spp. oocysts was detected by the McMaster floatation technique [32].

### 2.3. Statistical Analyses

All statistical analyses were performed using the statistical software package R 4.1.2. [33]. Firstly, the prevalence of each agent was obtained, and the possible influence of the vaccination status of the farm or the administration of halofuginone lactacte on the percentage of samples positive to BRoV, ETEC, BCoV and *C. parvum*, respectively, was analysed using chi-squared tests with Yates’ correction. Results were considered statistically significant at *p* < 0.05. Then, the samples were classified in those positive to a single agent and those positive to more than one enteropathogen. Finally, the number of different combinations of pathogens and the thirty most frequent ones were determined. The graphical representation of the data was performed using Microsoft Excel 2021 (Microsoft Corporation, Redmond, WA, USA) and the R package *ggplot2* 4.0.0 [34].

## 3. Results

### 3.1. Prevalence of the Target Enteropathogens

**Figure 1:**
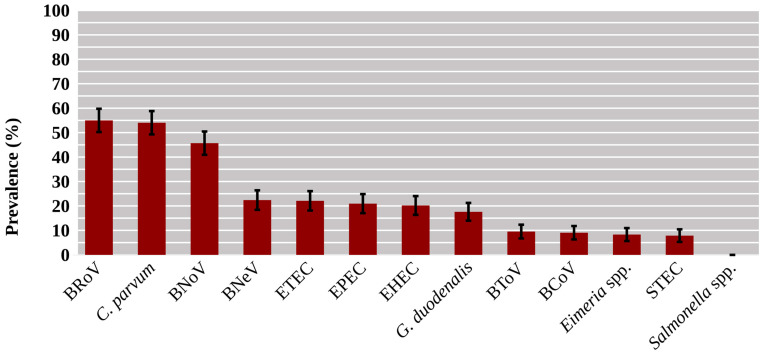
Prevalence of enteropathogens in diarrhoeic neonatal calves under one month of age from north-western Spain. BCoV: bovine Coronavirus; BNeV: bovine Nebovirus; BNoV: bovine Norovirus; BRoV: bovine Rotavirus A; BToV: bovine Torovirus; *C. parvum*: *Cryptosporidium parvum*; EHEC: enterohaemorrhagic *Escherichia coli*; EPEC: enteropathogenic *Escherichia coli*; ETEC: enterotoxigenic *Escherichia coli*; *G. duodenalis*: *Giardia duodenalis*; STEC: shiga-toxin producing *Escherichia coli*. Whiskers represent the 95% confidence interval.

Overall, 97.1% (408/420) of the samples were positive to at least one of the agents included in the study. BRoV, *C. parvum*, and BNoV were especially frequent, with prevalence rates ranging from 45.7% to 55.0% (Figure 1). Three diarrhoeagenic *E. coli* pathotypes (ETEC, EPEC and EHEC), BNeV and *G. duodenalis* were detected in around 20% of the samples, whereas the prevalence of the remaining studied agents was lower than 10%. *Salmonella* spp. was not isolated in any sample (Figure 1).

Vaccination against BRoV, ETEC and BCoV was performed in 33.3% (74/222) of the farms. Calves from vaccinated farms showed lower prevalences of BCoV (8/128; 6.3%; 95%CI 2.1–10.4% vs. 30/292; 10.3%; 95%CI 6.8–13.8%) and ETEC (15/128; 11.7%; 95%CI 6.2–17.3% vs. 78/292; 26.7%; 95%CI 21.6–31.8%), but a slightly higher prevalence of BRoV than those from unvaccinated ones (72/128; 56.3%; 95%CI 47.7–64.8% vs. 159/292; 54.5%; 95%CI 48.7–60.2%). Differences were only significant for ETEC (χ^2^ = 10.751; *p* = 0.001). Finally, treatment with halofuginone lactate for the prevention of cryptosporidiosis was implemented in the 21.6% (48/222) of the farms, and the prevalence of *C. parvum* in treated calves was slightly lower than in the untreated group (20/39; 51.3%; 95%CI 35.6–67.0% vs. 203/381; 53.3%; 95%CI 48.3–58.3%), but these differences were not significant (*p* > 0.05).

### 3.2. Analysis of Co-Occurrence Between Pathogens

**Figure 2:**
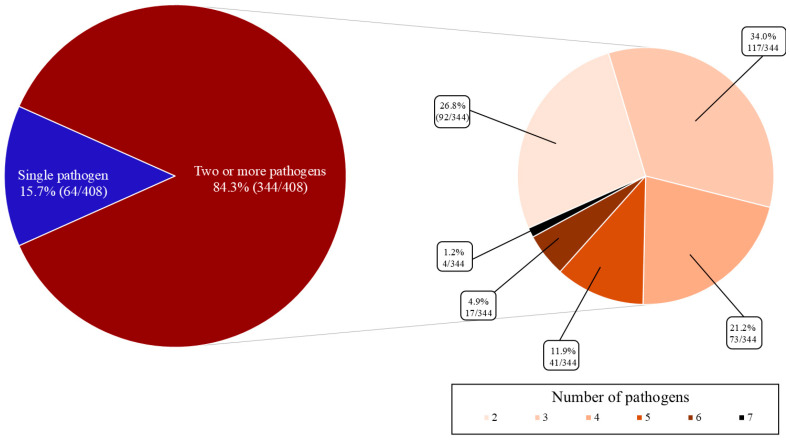
Co-occurrence of pathogens in positive faecal samples of diarrhoeic neonatal calves under one month of age from north-western Spain.

The detection of several enteropathogens (84.3%; 344/408) was more frequent than the identification of a single agent (15.7%; 64/408); concomitant presence of two, three and four pathogens were predominant, although up to seven pathogens were identified (Figure 2).

**Figure 3:**
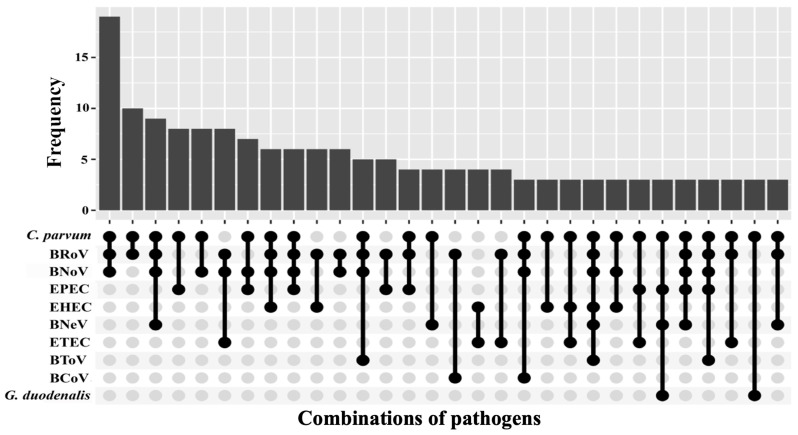
Thirty most frequent combinations of pathogens in diarrhoeic neonatal calves under one month of age from north-western Spain. BCoV: bovine Coronavirus; BNeV: bovine Nebovirus; BNoV: bovine Norovirus; BRoV: bovine Rotavirus A; BToV: bovine Torovirus; *C. parvum*: *Cryptosporidium parvum*; EHEC: enterohaemorrhagic *Escherichia coli*; EPEC: enteropathogenic *Escherichia coli*; ETEC: enterotoxigenic *Escherichia coli*; *G. duodenalis*: *Giardia duodenalis*.

A total of 172 different combinations of agents were found in this study, showing the thirty most frequent in Figure 3. The most common co-occurrences were those only comprising *C. parvum* and one or several viral agents (20.9%; 72/344 of associations). Thus, *C. parvum*, BRoV and BNoV constituted the most common pathogen combination in this study, and together with BNeV, the third most abundant one. In this regard, the presence of the emergent caliciviruses BNoV and BNeV in these combinations is remarkable.

**Figure 4:**
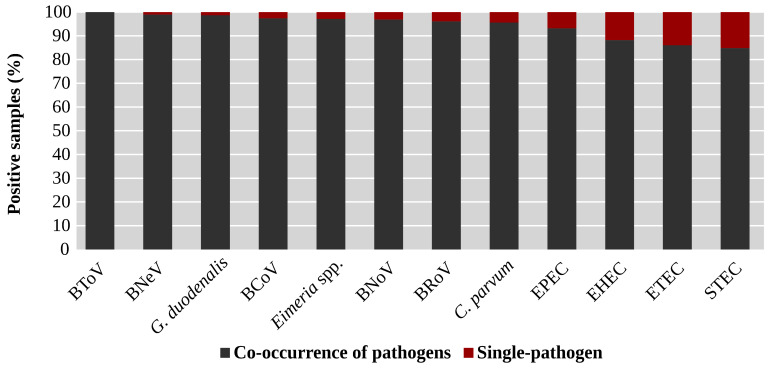
Distribution of samples positive to each enteropathogen according to whether they correspond to the presence of a single or different pathogens. BCoV: bovine Coronavirus; BNeV: bovine Nebovirus; BNoV: bovine Norovirus; BRoV: bovine Rotavirus A; BToV: bovine Torovirus; *C. parvum*: *Cryptosporidium parvum*; EHEC: enterohaemorrhagic *Escherichia coli*; EPEC: enteropathogenic *Escherichia coli*; ETEC: enterotoxigenic *Escherichia coli*; *G. duodenalis*: *Giardia duodenalis*; STEC: shiga-toxin producing *Escherichia coli*.

All the studied agents were detected in combination with other enteropathogens in more than 82% of the samples where they were detected (Figure 4). This was especially frequent in the case of BToV, identified only associated with other agents. Moreover, concurrent presence of pathogens was detected in 98.9% (93/94) and 98.7% (73/74) of the samples positive for BNeV and *G. duodenalis*, respectively (Figure 4).

## 4. Discussion

This study provides data on the prevalence of thirteen enteropathogens and their combinations in diarrhoeic calves under one month of age. It represents an important contribution to this field given the limited number of publications analysing such a wide range of pathogens in calves with diarrhoea. Our findings show that the presence of enteropathogens and their interactions play a key role in the onset of NCD, since at least one enteric agent was identified in a vast majority of the analysed samples (97.1%), and many samples contained multiple pathogens.

The most commonly detected pathogens in our study were BRoV and *C. parvum*, agreeing with most investigations worldwide and confirming that both enteropathogens are currently the major agents involved in NCD [14,16,35,36,37,38,39]. The prevalence of BRoV in this study (55.0%) was slightly higher than that previously obtained in diarrhoeic calves from Spain (42.7–50.6%) [18,20,40], whereas that of *C. parvum* (53.1%) was similar to the results reported in Spain in the last 25 years (49.2–64.7%) [18,20,41,42,43,44]. These high prevalences are surprising considering the availability of vaccines and commercial treatments aimed at preventing diarrhoea caused by both pathogens, and whose administration is common in cattle farms [45]. In this regard, in our study, the use of halofuginone lactacte for treating cryptosporidiosis resulted in a slightly lower prevalence of *C. parvum*. Nevertheless, it is worth noting that halofuginone lactate has a cryptosporidiostatic activity [46], and thus, although it is effective in delaying the onset of diarrhoea, and reducing mortality and oocyst excretion intensity, it does not prevent infection, which could be why the prevalence rates of *C. parvum* in treated and untreated animals were not significantly different. This could also explain why the prevalence of *C. parvum* in diarrhoeic calves in Spain has remained stable in the last 25 years despite the growing use of this treatment. In the same vein, our data revealed that vaccination against BRoV did not reduce its presence significantly and, in fact, a slightly higher prevalence of BRoV was observed in vaccinated farms. This finding does not imply that the available BRoV vaccines are not effective, since they reduce the incidence of diarrhoea caused by BRoV and its fatal consequences, but they do not completely prevent infection [47,48]. In addition, it has been demonstrated that antibodies transferred through colostrum from vaccinated dams bind to BRoV, as well as to BCoV and ETEC, preventing adhesion and invasion of enterocytes. These neutralized antibodies pass the intestinal tract and thus, they can be detected in faeces [49]. The agents then pass the intestinal lumen and are excreted with the faeces. In this regard, the onset of diarrhoea in some of the studied vaccinated animals could be due to the concurrent presence of other pathogens that constitute the underlying cause, especially considering that other agents were detected in the 96.1% of the samples positive to BRoV. However, since these vaccines are administered to the dams before delivery, detection of positive animals may also be related to deficiencies in colostrum management [50]. Finally, it is also worth noting that the noticeable presence of BRoV in diarrhoeic calves might also be related to the emergence of new viral strains able to evade the vaccinal immune response [18,40,51,52,53,54], reinforcing the need for further studies on the molecular characterization of this virus.

At least one *E. coli* diarrhoeagenic pathotype was detected in more than half of the samples. Enterotoxigenic *E. coli* (ETEC), the only pathotype clearly related to NCD [55], was predominant, with a prevalence rate higher than those previously reported in Spain (4.1–8.0%) [20,21], although it must be considered that the serological methods used in previous investigations are less sensitive than the molecular techniques used in our study [56]. To mitigate the impact of ETEC on neonatal calves, many farms have implemented preventive measures such as vaccination [57]. In this study, the introduction of vaccination programs resulted in a significant lower prevalence of this pathogen. Therefore, adopting vaccination strategies is strongly advisable in farms where ETEC-related NCD cases have been detected or where risk factors suggest a potential outbreak.

This investigation provides the first data on the presence of emerging viruses in diarrhoeic neonatal calves from Spain, showing that BNoV and BNeV are prevalent, whereas the presence of BToV in diarrhoeic neonatal calves is limited. It is worth noting that, generally, our prevalence rates are noticeably higher than those previously obtained for BNoV (8.9–34.2%), BNeV (7.0–13.1%) or BToV (3.6–11.1%) in diarrhoeic calves from Europe [58,59,60,61,62,63,64,65,66]. In addition, considerable prevalence rates of EPEC and EHEC (21.0% and 20.2%, respectively) were detected, being comparable to that of ETEC and greater than those previously described in Spain (8.0–9.0%) [67]. Similarly, the presence of *G. duodenalis* was common among diarrhoeic suckling calves, showing a higher prevalence than those previously reported (3.5–10.6%) in diarrhoeic calves from Europe [6,41,68]. It is worth noting that the role of BNoV, BNeV and BToV, as well as that of EHEC, EPEC and *G. duodenalis* in NCD has recently been questioned in a number of studies, since these agents have also been identified in a substantial proportion of asymptomatic animals [62,69,70,71,72,73,74,75,76]. Therefore, and considering the noticeable prevalences of BNoV, BNeV, EHEC, EPEC and *G. duodenalis* in diarrhoeic suckling calves detected in this investigation, further research is needed to unravel their role in NCD. This information is the key for assessing the potential need to include these agents in NCD routine diagnostic panels and to develop vaccines against them. Another significant finding of our study was the low prevalence of BCoV, which is consistent with previous research conducted in Europe, where prevalence rates varied from 1.0% to 23.6% [14,18,20,21,39,77,78]. All this evidence suggests a significant reduction in the relevance of BCoV in NCD, probably due to the widespread implementation of effective vaccination strategies [48].

Moreover, our data revealed that *Eimeria* spp. was not common. Coccidial infections are especially frequent in animals between 3 weeks and 6 months of age [6,79], which may explain the low prevalence detected in our study, as calves up to 30 days old were exclusively included. In addition, the prevalence of STEC was low and in line with previous observations indicating a global decreasing trend in the significance of this pathotype in calves [72]. No positive samples to *Salmonella* spp. were detected; these results could be due to the absence of a pre-enrichment step prior to culturing in XLD agar, as recommended in ISO 6579-1:2017 [80], which could result in an underestimation of the presence of this bacterium. Nevertheless, if NCD was caused by *Salmonella* spp., bacterial shedding in faeces should be high enough to allow detection without needing that pre-enrichment step.

It must be considered, as a limitation of our study, that pathogens were only detected by laboratory methods without confirmation of their pathogenic effect in the animal. As stated before, the role of some of the analysed enteropathogens in NCD is still controversial [11,81], and other methods, such as histopathology or immunohistochemistry, or including healthy control animals, would be needed to confirm actual infection. In this regard, the inclusion of non-diarrheic animals as controls could have been useful; however, healthy control animals from diarrhoeic farms were not included in our study because each calf was only sampled once, which hinders its suitability as a control, given that it is unknown whether it later developed diarrhoea and it has been reported that these control calves might show diarrhoeic faeces shortly after sampling [14,82].

Although a thorough analysis of available data suggests that most NCD outbreaks are the result of the infection with several agents [2,21,37], more complete and updated information is needed. In this context, our results provide novel and relevant information on the aetiology of NCD, revealing that the concurrent presence of agents is predominant in diarrhoeic calves under one month old and showing a substantially higher percentage of concurrent pathogens (84.3%) than those previously described in Spain (15.7–42.5%) [18,20,21] and other European countries (19.7–32.6%) [6,14,81]. It must be considered that those aforementioned studies only examined four or five pathogens. When studies including a broader spectrum of agents were evaluated, our data also show a higher prevalence of associations between agents. Thereby, Cho et al. [11] analysed the presence of 11 pathogens in diarrhoeic calves mostly under 90 days of age, detecting 55.0% of mixed infections. Similarly, Lee et al. [17] analysing fourteen different agents, reported 42.1% of co-infections in diarrhoeic calves aged less than 7 months. Moreover, it is worth noting that other agents potentially involved in NCD, such as bovine astrovirus or bovine enterovirus [1] were not included, suggesting that the percentage of co-infections could be even higher.

Focusing on the number of agents involved, the high frequency of the concurrent presence of two agents is in line with the previous literature (15.7–42.4%) [6,11,14,15,17,18,20,21]. However, the prevalence of triple and quadruple infections was higher than those reported in earlier studies, even those analysing a larger number of pathogens (triple: 5.5% and 16.0%; fourfold: 3.7% and 5%) [11,17]. In agreement with our findings, both Cho et al. [11] and Lee et al. [17] identified a small percentage of infections involving more than four agents (3.0% and 0.6%, respectively), but none of them detected seven agents in a single sample.

The presence of each analysed enteropathogen varied according to its co-occurrence with other pathogens. The agents most frequently identified as single pathogens were STEC, ETEC and EHEC, which may be related to their onset at a very early age since several studies have detected the highest prevalence rates of these agents in diarrhoeic calves under a week of age [79,83,84,85,86,87]. Nevertheless, the detection of pathogens that were not excreted in faeces at the time of sampling due to longer incubation or prepatent periods cannot be ruled out. In contrast, viral agents, particularly BNoV and BNeV, and *C. parvum* were especially involved in the most frequently detected combinations. The frequent association between these caliciviruses and *C. parvum* might be due to a viral immunosuppressive effect favouring the development of the protozoan’s life cycle [11] or to their different pathogeneses. These caliciviruses induce lesions in more proximal segments of the small intestine than *C. parvum*, which mainly parasitizes enterocytes from the ileum and proximal portions of the large intestine [10,88]. Thus, *C. parvum* infections would reduce water reabsorption, impairing the compensation for the losses due to the lesions caused by caliciviruses in more proximal segments and contributing to the onset of diarrhoea. This would explain why the prevalence of BNoV and BNeV as single pathogens was low in this study (3.1% for BNoV; 1.1% for BNeV) and would be in line with the results of previous investigations which reported higher prevalences of these caliciviruses in asymptomatic animals [62,69,70]. Nevertheless, a significant association between the presence of BNoV or BNeV and the onset of diarrhoea has also been reported [11,12,63]. Therefore, further research is needed to determine the role of these agents in mixed infections that lead to the onset of NCD.

## 5. Conclusions

Our results demonstrate that the aetiology of NCD is extremely complex. Thus, most diarrhoeal cases in neonatal calves from north-western Spain are caused by the concurrent presence of several (up to seven) agents. Associations between *C. parvum* and viruses such as BNoV, BRoV and BNeV predominated. This fact might be due to a viral immunosuppressive effect favouring the development of *C. parvum*. Moreover, the significant presence of BNoV, BNeV, EHEC, EPEC and *G. duodenalis* in diarrhoeic calves up to 30 days old suggests that these agents may play a role in the onset of clinical signs. Therefore, their inclusion into routine diagnostic panels (usually limited to ETEC, BRoV, BCoV and *C. parvum*) may enhance the aetiological diagnosis of NCD, which is essential for implementing the most effective control measures.

Nevertheless, since NCD is a multifactorial syndrome, optimizing control strategies on farms also relies on thorough anamnesis to identify the risk factors involved in the emergence of outbreaks.

## Data Availability

The original contributions presented in this study are included in the article/supplementary material. Further inquiries can be directed to the corresponding author.

## Supplementary Materials

The following supporting information can be downloaded at: https://www.mdpi.com/article/10.3390/ani15182735/s1, Table S1: Characteristics of the vaccines against neonatal calf diarrhoea associated with bovine rotavirus (BRoV), bovine coronavirus (BCoV) and Enterotoxigenic *Escherichia coli* available in Spain during 2017–2020.
