# Supplementary material for: Importance and Characterisation of Concurrent Pathogens in Diarrhoeic Calves from North-Western Spain
Source: Animals (Basel). 2025 Sep 19;15(18):2735. doi: 10.3390/ani15182735 (PMC12466406; doi:10.3390/ani15182735)
Supplement: Supplementary file 1 [file animals-15-02735-s001.zip › animals-3788062-supplementary.pdf]

Table S1. Characteristics of the vaccines against neonatal calf diarrhoea associated with bovine rotavirus (BRoV), bovine coronavirus (BCoV) and Enterotoxigenic *Escherichia coli* available in Spain during 2017-2020 .

Vaccine	Pathogen Strain
Bovilis Rotavec Corona (Merck Sharp & Dohme Animal Health, S.L., Salamanca, Spain)	BRoV serotype G6P5, strain UK-Compton, inactivated<br>BCoV, strain Mebus, inactivated<br>*E. coli* serotype O101:K99:F41, strain CN7985, fimbrial adhesins F5 and F41, inactivated
Bovisan Diar (FORTE Healthcare Ltd., Dublin, Ireland)	BRoV serotype G6P1, strain TM-91, inactivated<br>BCoV, strain C-197, inactivated<br>*E. coli* strain EC/17, fimbrial adhesins F5 and F41, inactivated
Scourguard 3 (Zoetis, Louvain-La-Neuve, Belgium)	BRoV serotype G6P1, strain Lincoln, live attenuated<br>BCoV, strain Hansen, live attenuated<br>*E. coli* strain NADC 1471, fimbrial adhesin F5, inactivated
